# Doing ‘detective work’ to find a cancer: how are non-specific symptom pathways for cancer investigation organised, and what are the implications for safety and quality of care? A multisite qualitative approach

**DOI:** 10.1136/bmjqs-2024-017749

**Published:** 2025-01-29

**Authors:** Georgia B Black, Brian D Nicholson, Julie-Ann Moreland, Naomi J Fulop, Georgios Lyratzopoulos, Ruth Baxter

**Affiliations:** 1 Wolfson Institute of Population Health, Queen Mary University of London, London, UK; 2 Applied Health Research, University College London Research Department of Epidemiology and Public Health, London, UK; 3 Nuffield Department of Primary Care Health Sciences, University of Oxford, Oxford, UK; 4 Department of Radiology, Oxford University Hospitals NHS Foundation Trust, Oxford, UK; 5 Department of Behavioural Science and Health, University College London, London, UK; 6 Cambridge Centre for Health Services Research, University of Cambridge, Cambridge, UK; 7 Department of Epidemiology & Public Health, University College London, London, UK; 8 University of Leeds Faculty of Medicine and Health, Leeds, UK

**Keywords:** Diagnostic errors, Health services research, Qualitative research, Patient Safety

## Abstract

**Background:**

Over the past two decades, the UK has actively developed policies to enhance early cancer diagnosis, particularly for individuals with non-specific cancer symptoms. Non-specific symptom (NSS) pathways were piloted and then implemented in 2015 to address delays in referral and diagnosis. The aim of this study was to outline the functions that enable NSS teams to investigate cancer and other diagnoses for patients with NSSs.

**Methods:**

The analysis was derived from a multisite ethnographic study conducted between 2020 and 2023 across four major National Health Service (NHS) trusts. Data collection encompassed observations, patient shadowing, interviews with clinicians and patients (n=54) and gathered documents. We used principles of the functional resonance analysis method to identify the functions of the NSS pathway and analyse their relevance to patient safety.

**Results:**

Our analysis produced 29 distinct functions within NSS pathways, organised into two clusters: pretesting assessment and information gathering, and post-testing interpretation and management. Safety-critical functions encompassed assessing the reason for referral, deciding on a plan of investigation and estimating the remaining cancer risk. We also identified ways that teams build and maintain safety across all functions, for example, by cultivating generalist-specialist expertise within the team and creating continuity through patient navigation. Variation in practice across sites revealed targets for an NSS pathway blueprint that would foster local development and quality improvement.

**Conclusions:**

Our findings suggest that national and local improvement plans could differentiate specific policies to reduce unwarranted variation and support adaptive variation that facilitates the delivery of safe care within the local context. Enhancing multidisciplinary teams with additional consultants and deploying patient navigators with clinical backgrounds could improve safety within NSS pathways. Future research should investigate different models of generalist-specialist team composition.

**WHAT IS ALREADY KNOWN ON THIS TOPIC**

- Non-specific symptom (NSS) pathways were introduced in England as novel services to address delays in referrals and diagnoses for patients with non-specific cancer symptoms. These pathways involve a battery of tests and consultant-led diagnostic management.

**WHAT THIS STUDY ADDS**

- Our findings outline the various functions required to look for cancer in patients with NSSs and how the functions fit together to create a system for cancer investigation and management. It sheds light on the complex work of patient assessment, testing and risk.
- We have identified safety-critical functions within these pathways, such as assessing patient suitability, tracking test results and ensuring continuity of care.
- The study highlights the significance of creating generalist healthcare teams for cancer diagnostics with specialised skills that are capable of investigating NSSs safely and fostering learning environments to develop the necessary expertise.

**HOW THIS STUDY MIGHT AFFECT RESEARCH, PRACTICE OR POLICY**

- Our findings suggest that policymakers should focus on fostering the conditions that facilitate the delivery of safety-critical functions within NSS pathways that allow for local learning, development and generalist-specialist skill acquisition.

## Introduction

 Over the past two decades, the UK has developed a cancer diagnosis policy programme to align outcomes with other high-income countries.^1^ This programme includes quality indicators such as rapid referral-to-treatment intervals and standardised investigative pathways.^2^ Service improvement has focused on a number of indicators of poor delivery, including emergency cancer presentations, multiple primary care consultations before referral and referrals to multiple specialties before diagnosis.^3^,^5^

Around half of the patients with cancer initially experience non-specific symptoms (NSS), causing delays in referral, investigation and diagnosis.^6^ These include site-specific symptoms such as coughing and constipation, as well as non-site-specific signs like fatigue and weight loss. Previously, patients with non-site-specific symptoms often experienced diagnostic delays due to the undifferentiated nature of the symptoms, and the need for multiple suspected cancer referrals before diagnosis.^7^,^9^ To meet this need, the National Health Service (NHS) long-term plan introduced novel services in the UK known as NSS pathways.^10^ These are urgent referral services based on the Danish three-legged model,^11^ which includes a pathway for specific alarm symptoms, ‘No-Yes Clinics’ for low-risk-but-not-no-risk symptoms and a pathway for non-specific serious symptoms. The latter services are designed to determine the cause of NSSs rather than solely ruling out specific cancers. Similar pathways have been developed in Norway and Sweden.^12^ Patients undergo faecal and blood testing in primary care, followed by comprehensive assessments, imaging and consultant-led diagnostic management involving different medical specialties in secondary care.^13^,^15^ Patients are either referred onward to specialist services for further investigation and treatment or discharged to primary care with management advice where appropriate. National guidance emphasises the importance of avoiding multiple fragmented referrals and taking a holistic approach to diagnosis in NSS pathways.^14^

Evaluative research on these services is emerging from Denmark, Norway, Sweden and the UK,^1213 15^,^26^ suggesting that they can shorten the time from referral to diagnosis but also reveal wide variation in design and function. Cancer detection rates are also promising, with an average incidence rate of 7%–12%,^1327^,^29^ although rates are higher in Denmark (11%–16%) due to differences in patient referral criteria.^7 22 30^ The most commonly detected cancers are colorectal, haematological, lung and pancreatic.^13 22^

In a previous qualitative paper, we highlighted difficulties in delivering NSS pathways consistently due to ambiguities in policy and guidance (in press—to be added ASAP). For example, some NSS teams implemented the pathway as a means to swiftly exclude cancer diagnoses, whereas others sought to provide a comprehensive service addressing NSSs beyond cancer. Meeting national timed targets for cancer within financial constraints encouraged pathway development towards the more restrictive approach and exacerbated uncertainty about the service’s primary objectives and responsibilities. In a different qualitative publication, we also highlighted missed opportunities for organisations to support patient understanding during their investigations in NSS pathways, which could lead to delays, overtesting and inappropriate help-seeking postdischarge.^31^

NSS pathways have similar goals to other cancer pathways in terms of timely diagnosis but must do this in the absence of a clear underlying cause for symptoms, resulting in a potential investigation of multiple organ systems. The aim of this paper is to encourage consistent and effective service development by:

- Identifying the functions that enable NSS teams to investigate cancer and other diagnoses for patients with NSSs.
- Identifying performance variation in safety-critical functions.
- Describing team dynamics that affect system safety and quality of care.

## Methods

This paper presents data from a multisite ethnographic study conducted across four acute NHS trusts in the southeast of England between 2020 and 2023. All four trusts were in urban or suburban settings, with similar diagnostic tests available. They varied in size from 421 to 1770 inpatient beds. Three were teaching hospitals. Population demographics varied widely; for example, the percentage of local residents who were white ranged from 27% to 77%, and male life expectancy ranged from 79.58 to 82.15 years. Data collection focused on services providing cancer investigation for patients with NSSs, referred to as ‘rapid diagnosis centre’ or ‘non-specific symptom pathway’ as outlined by NHS England’s 2019 service specification, updated in 2022.^32 33^ Reporting follows the Standards for Reporting Qualitative Research (see online supplemental appendix).^34^

### Data collection

We collected several types of data at each site, including observation, patient shadowing, interviews with clinicians and patients and document analysis related to pathway implementation. The study ran concurrently at all four sites, with the researcher spending prolonged time with each team and attending their regular meetings.

#### Observation

After obtaining consent to observe staff activities, one researcher with extensive qualitative training and experience conducted approximately 44 hours of observations across the four sites. This was heavily restricted by COVID-19 working practices. This included virtual staff meetings, shadowing service staff, attending clinics and recording open-ended notes in notebooks. These notes were later refined for clarity using electronic software. Particular attention was paid to activities, such as patient clinics and assessments, team meetings, triage, discharge meetings and imaging/testing procedures. Patients present during observations were verbally informed about the research, and their details were not recorded. We conducted observations within the different environments relevant to the NSS team. The environments differed for each trust due to the different configurations of the service, office and clinic rooms, specialties involved in patient care and COVID-related restrictions during the data collection period. We did not conduct any observations with teams outside the NSS pathway.

#### Patient shadowing

Clinical team members approached patients who were selected to promote diversity in age, gender, sociodemographics and ethnicity (see table 1). Patients were asked for consent to be contacted by the researcher, given study materials, and then provided written or audio-recorded consent. 27 patients were interviewed, and some allowed the researcher to shadow them during hospital appointments, either through postappointment phone calls or by accompanying them in person. 11 patients declined participation after initially consenting.

**Table 1:** Patient and professional participant demographics

**Patient demographic characteristics (n=27)**	
Sex	
Female	13
Male	14
Age	
Mean	67.6
Range	35–94
Education	
None	3
O level or GCSE/equivalent	3
A level or higher	5
Higher education qualification below degree level	9
Degree	6
PhD	1
Employment	
Working full time	8
Working part time	5
Retired	14
Ethnicity	
White British	17
White (other)	2
Black African	1
Asian	3
Asian British	1
Black Caribbean	1
Marital status	
Single	5
Married	14
Divorced or separated	3
Widowed	5
Index of multiple deprivation quintile by postcode	
1 (most deprived)	1
2	6
3	6
4	8
5 (least deprived)	6
**Professional demographic characteristics**	
Professional role	
Consultant	9
Nurse	4
Navigator	3
Cancer alliance/commissioners	9
Policy maker	2
Organisational context	
Site 1	3
Site 2	2
Site 3	4
Site 4	8
National	3
Cancer alliance 1	4
Cancer alliance 2	3

#### Interviews with clinicians and other stakeholders

All staff within the NSS pathways were invited for interviews and identified through direct encounters during observations or referrals from colleagues. Due to the nature of the services, this included multiple specialties. Additionally, relevant staff from local cancer alliances, integrated care boards and national cancer teams were approached. Out of 35 invited, 27 agreed to participate. Semistructured interviews were conducted about their experiences of NSS pathways following a flexible topic guide developed by the authors. Two participants were interviewed together.

#### Documents

We searched government websites and search engines for documents related to early cancer diagnosis and NSS pathways through an exploratory and non-exhaustive process. Local teams provided additional materials such as patient-facing documents and audit reports by request.

### Analysis

We employed multiple analytic strategies, including writing and rewriting observational field notes, inductive coding of interviews, document and observation data and research group discussions. Audio-recorded data (interviews, shadowing conversations) were transcribed verbatim and anonymised. All field notes, transcriptions and documents were treated as data.

Analysis began in parallel with fieldwork. We initially reviewed field notes to support discussion and exploration of issues for further data collection. We completed inductive coding of observation notes facilitated by NVivo qualitative software. Using an iterative approach, we engaged in discussions to identify differences between sites, with a particular focus on aspects affecting patient safety.

We used some principles of the functional resilience analysis method (FRAM), which provides a way of analysing how everyday performance is delivered within complex sociotechnical systems. FRAM facilitates a description of work activities as they are actually performed rather than as they are ‘imagined’, for example, in policy documents or service specifications.^35^ FRAM models are developed by identifying the key activities or ‘functions’ that exist within a particular system and then examining the variation within each function, the relationships (couplings) that exist between them, and the resonance that emerges within the system as a result of this. We chose to apply principles of FRAM methodology to the NSS pathway because these are newly implemented services lacking established guidelines or evidence-based practices. As such, they exhibit a broader scope and greater operational variability compared with more established, site-specific cancer pathways. FRAM allows for a nuanced examination of functional interactions, capturing the variability inherent in an evolving service where roles, systems and processes are still under development. This approach is particularly well-suited to identifying emergent risks and adaptive behaviours in a nascent, less standardised context compared with other methods that capture linear, sequential processes such as swim lanes or process mapping.^36^

In this study, we defined the system boundary to include functions that existed within the hospital NSS service (ie, we excluded functions conducted in primary care or patients’ homes). FRAM provided a structured way to interpret our findings following a three-step process. First, we identified the key functions that existed across all sites from our initial inductive coding and represented them in a tabular format. ‘Foreground functions’, the central focus of a FRAM analysis, were those that were critical to understanding how the service achieves patient care.^37^ We also derived ‘background functions’, which are cross-cutting or supportive of these foreground functions and provide necessary context but are not the immediate focus of the study. Second, we identified and documented descriptions of variation in relation to how each of these functions was carried out within and between sites, comparing summaries and verbatim data. Third, we explored couplings between functions to iteratively develop a visual model of the NSS system. This was done primarily by considering two aspects of each function: the ‘input’, which activates or starts a functional activity and the ‘output’ that results from it. Due to the broad scope of the NSS pathway and the need to represent care delivery at all four sites, we maintained a high level of abstraction to retain usability and clarity in the resulting system model. When creating the model, we therefore described the more basic input-output relationships between functions and did not model other aspects of each function, such as preconditions, resources, control and time. Our resulting system model is also presented in a more accessible way rather than displaying it via hexagons as is typical with FRAM.

Clinical authors provided feedback and edited the identified functions and overall model. Through these discussions and in reference to the number of inputs and outputs on the emergent model, we identified that certain functions had a more significant safety impact. We focused on these, in particular, to explore variation in delivery and consider the potential effects on other interdependent functions. This was an unforeseen aspect of the analysis.

## Findings

### Context

The four NSS pathways were organised differently, with a variety of clinical specialties and roles/responsibilities. They had distinct organisational qualities, such as the environments in which they made contact with the patient, including virtual and in-person clinics. They also had many common characteristics, such as the type of symptoms described by patients, which were predominantly weight loss and fatigue. Nearly all the sites deployed a CT scan of the chest, abdomen and pelvis as the first-line investigation. Many patients had a complex history with respect to their symptoms and had often had several recent investigations. Most patients that we interviewed had experienced fatigue or weight loss, although this was often not the reason that they initially sought help from their general practitioner (GP) (for more details, please see our previous paper where this is discussed in more detail).^31^ All four NSS teams relied on a network of contacts within the hospital to support a range of potential diagnoses, and all sites pursued cancer and non-cancer investigations. There was a high proportion of incidental findings in all services.

### Describing the functions within the NSS pathway

**Figure 1:**
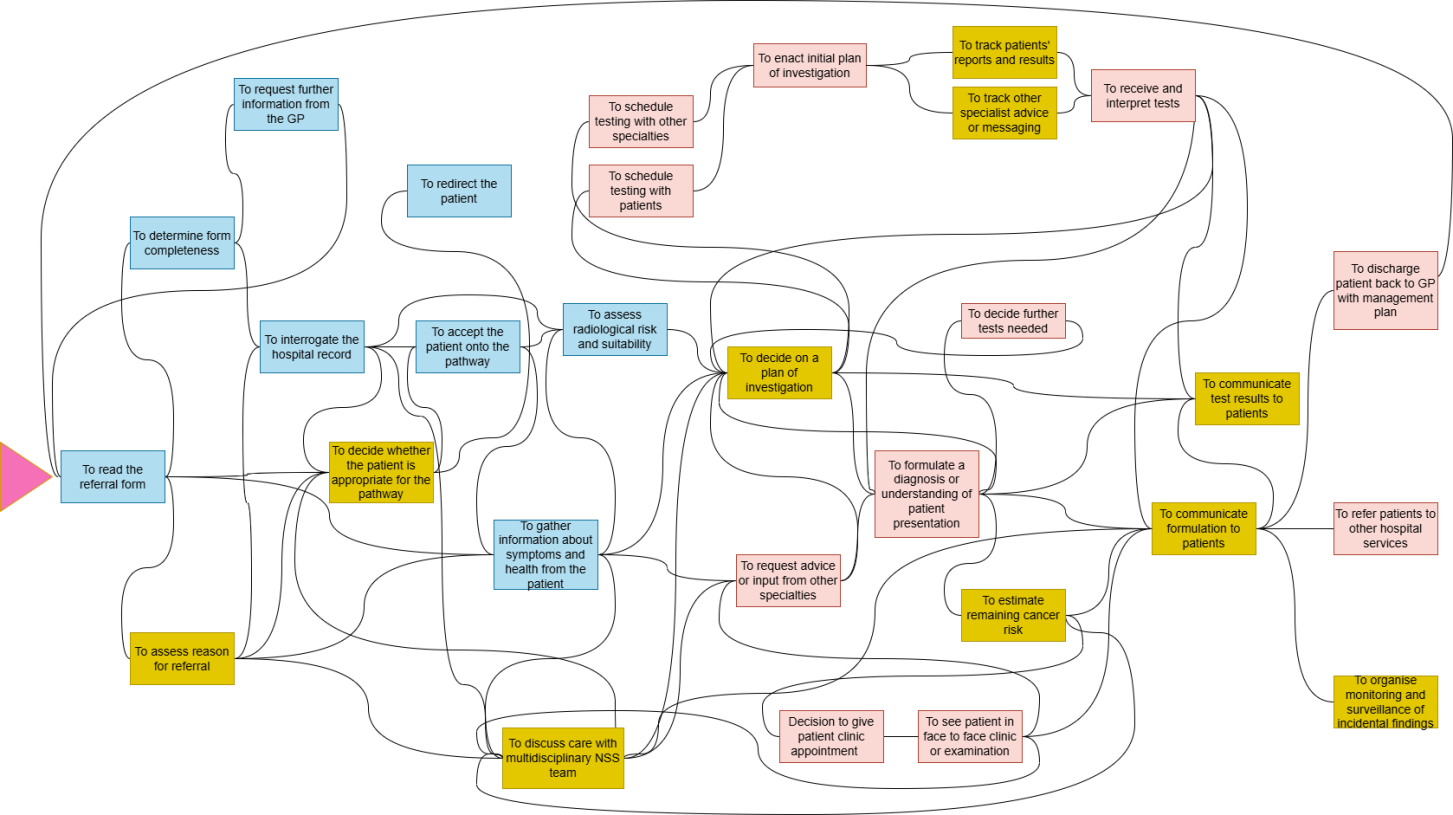
Functional system visualisation of the non-specific symptom pathway. Note: Blue functions denote Cluster 1; pink functions denote Cluster 2, and yellow functions denote safety-critical functions.

Our initial analysis identified 20 discrete foreground functions within the NSS pathway. After discussion within the research team and with our clinical authors, additional functions were added by splitting some functions and reinterpreting those that had been derived through analysis. The lines represent inputs and outputs. The final system diagram includes 29 functions (see figure 1). These functions can be broadly grouped into two clusters that are outlined below.

1. Pretesting assessment and information gathering (blue).
2. Post-testing interpretation and management (pink).

Within these clusters, 10 functions were identified as being safety-critical (yellow). The pink triangle indicates the entry point into the system for most patients, which is the receipt of a referral letter from primary care. The diagram is not chronological, and multiple functions can occur simultaneously depending on their interdependency. There is no definitive final function, as there were significant differences in discharge and long-term follow-up between the sites; however, the general flow of functions is left to right.

#### Cluster 1: pretesting assessment and information gathering

The pathway began with the receipt of a referral form from the GP. Clinicians assessed the completeness of the form to determine the reason for referral. They also reviewed relevant medical history from hospital records and evaluated the patient’s suitability for the pathway; some referrals were redirected to an alternative pathway if clinically indicated, for example, the urgent suspected colorectal cancer pathway.

Once accepted onto the pathway, clinicians collected additional information about the patient’s symptoms through either a face-to-face clinic or a telephone appointment. The NSS team then collaboratively decided on the most appropriate investigations, occasionally seeking advice from specialist colleagues.

The decision-making process often involved what NSS staff described as a substantial amount of ‘detective work’ to explore the underlying cause of the patient’s symptoms. For example, this could include rereading the GP referral form, which was often more than 10 pages long, requesting additional information from the GP, scrutinising hospital records, particularly for past test results and investigations and speaking to patients. An example of ‘detective work’ is provided in box 1 based on the observation data.

Box 1 **Observation demonstrating ‘detective work’ (site 2)**

In one observation, the team prepared for a clinic meeting with a patient who had lost weight as indicated in the general practitioner’s (GP) referral letter. The team looked at the hospital patient record system to try and establish the patient’s normal weight, as a recent telephone assessment with the patient last week had led to further confusion about how much weight they had lost. The team discussed the patient being under recent stress and having recently had a chest X-ray and a battery of other tests. The team used six different record systems to search for information, including the electronic patient record, a radiology system, a clinic letter system, a system for reports from colonoscopy, ultrasound and other investigations and a summary of primary care records and blood test results.

This ‘detective work’ was often poorly documented, which had downstream ramifications if someone in the team needed to refer back to the information gathered at a later date. Staff members recorded information in different ways, including jotting down notes in a personal notebook or diary, composing a brief email to a colleague to request a specific action or using a standardised template within the electronic health record. Despite these efforts, the meticulously gathered details were often oversimplified or omitted, leading to redundant investigative work later in the patient’s care pathway. This resulted in duplicative work, particularly at the point of discharge when the information had to be gathered again to write the discharge letter.

#### Cluster 2: post-testing interpretation and management

Investigations were timetabled through arrangements with the patient and services within the hospital (eg, radiology). The navigator tracked whether these investigations were complete and whether a report had been written with the results. The navigator typically conveyed the results to the patient on the telephone, although this was done in a clinic in some settings. In consultation with the patient, a member of the NSS team created an explanation for the patient’s symptoms and decided whether the patient required further investigations or not. If a cancer was found, the patient was directed to a specialist team (eg, respiratory). If a non-cancer disease was found, the patient was either referred to another specialty or back to their GP for management. If no disease was found, the NSS team issued advice (including retesting and symptom monitoring) to the GP and patient.

A key aspect of NSS working was the ability to draw on a wide range of advice as part of investigating the patient. For example, the lead clinician or navigator could request additional clinical support (either formally or informally) from other specialties within the hospital, from the GP, from the other members of their team and from the patient themselves. Box 2 describes an example of this from interview data.

Box 2 **Interview description of drawing on a wide range of knowledge**

In an interview, Dr X described a 95-year-old patient who had undergone a CT scan, and the radiology report had diagnosed a bowel thickening. The report suggested that the patient has an oesophago-gastro-duodenoscopy, which is an invasive camera test of the oesophagus, stomach and small bowel. After discussion with the non-specific symptom team, Dr X was not entirely satisfied with this conclusion as it was not in keeping with the patient’s advanced age and asked gastroenterology colleagues for a second opinion. The gastroenterologists suggested that the patient could have a magnetic resonance enterography, which is a special type of resonance imaging and much less invasive than the camera test. The scan is done using contrast material to produce detailed images of the small intestine and bowel. This enabled Dr X to come up with an appropriate investigation plan for the patient.

Participants also described that this cluster of functions was dependent on the NSS team’s skills in connecting to other areas of the hospital. This was seen as both a state of mind (ie, being flexible to different interpretations of the patient’s symptoms) and being well connected through professional work. For example, one radiographer held the view that their pre-existing connections across the hospital from working in radiology were an advantage for seeking relevant support in the NSS pathway:

The other thing about radiographers is that you end up working with lots of clinical teams and that means that you know, oh I know someone in haematology I am just going to give them a call. Or I know this surgeon they might be able to help me and the problem is your signposting is a little bit more effective. And like, I know it’s really cliché to say, it’s not what you know it’s who you know, but in some cases it kind of is, particularly when you are trying to establish something like this. It’s helpful to have links in other places because it makes the sort of pathway of care a little bit easier. (RDC16, Navigator)

Other participants concurred that a key skill for NSS staff was seeing the interconnection between symptoms and considering creating non-standard diagnostic routes:

(NSS staff) know how to probe and kind of make those connections. Can work with their clinic colleagues from other clinical disciplines as and when required. So I guess both in terms of understanding but also the skill, key skill is basically having that kind of holistic viewpoint and being able to kind of build relationships with clinicians from other disciplines. Rather than being kind of a very fixed mentality about what might be wrong with the patient. (RDC01 Cancer Alliance)

### Safety-critical functions of the NSS pathway and performance variation within and between sites

In this section, we describe a subset of the foreground functions that we have identified as being safety-critical; these were identified through two criteria: (1) those with particularly numerous up and/or downstream effects and (2) indications by clinical authors. The safety-critical functions were:

- To assess the reason for referral.
- To decide whether or not the patient is appropriate for the pathway.
- To decide on a plan of investigation.
- To track patients’ reports and results.
- To track other specialist advice or messaging.
- To discuss the case with the multidisciplinary NSS team.
- To estimate remaining cancer risk.
- To communicate test results and formulation to patients.
- To organise monitoring and surveillance of incidental findings.

These safety-critical functions, in terms of their nature, observed variation within and between sites and data regarding upstream or downstream safety effects are described in table 2. Variation was ascertained through interview and observation data.

**Table 2:** Safety-critical functions of the NSS pathway, observed performance variation and data-elicited examples of safety effects

Function(s)	Observed variation between and within sites	Example of function and any upstream or downstream safety effects
To assess the reason for referral	Variation observed in the level of completeness of the referral form from GPs. Also wide variation in the amount of information in the narrative summary part of the form. All sites used a structured proforma to try to standardise the information provided.	(Site 2) Nurse X reviewed a referral form from a GP, trying to deduce the reason for the patient’s symptoms. She wondered if the GP was simply passing off their job to a specialist, as the referral form lacked crucial information about the patient’s diverticulitis. Nurse X mentioned that the best information comes from nurse-led patient assessments as referral forms often contain ‘lame information’. Meanwhile, Dr Y was preparing for a phone call to discuss the patient’s case. He struggled to find the necessary information in the referral form which would indicate whether the patient had any relevant pre-existing conditions. Downstream, this led to difficulties in deciding whether or not to accept the patient onto the pathway and making a plan of investigation.
To decide whether or not the patient is appropriate for the pathway	Deciding whether or not to accept the patient onto the pathway differed between the four sites. One team used a multi-disciplinary team setting for triage. In others, a consultant or navigator made the decision alone. There are divergent practices around handling (1) incomplete referral forms (2) ineligibility criteria and (3) diversion to other cancer pathways. These different approaches reflected the varying needs and resources of each hospital, as well as the importance of established procedures for patient referrals for suspected cancer.	(Site 1) Nurse A reviewed a referral for a patient who has had several transient ischaemic attacks, clear lungs on chest X-ray, groin pain and a hernia. Suspecting liver fibrosis or cirrhosis, she felt that hepatology should have taken the referral, but was not sure why they did not. She discussed the case with a consultant who suggested they accept the referral, and order additional tests like alpha-fetoprotein, full blood count and blood film and iron studies. Looking at the patient’s record, they found that the patient had already been reviewed by neurology and wondered why the GP did not mention this in their referral. Overall, Nurse A and her team felt that they made safer decisions for patients when they used a structured triage process, leading to a more efficient and safe plan of investigation.
To decide on a plan of investigation	NSS teams decided on a plan of investigation based on the information gathered in the referral and patient assessment. The plan depended on whether the patient had had other recent investigations at the same or a different hospital, which was often difficult to find out. This could include imaging, further blood testing or other outcomes. There was wide variation in the way decisions were made. At one site, the consultant made investigation decisions alone. At two sites, some decisions were made by a multidisciplinary team including a consultant, nursing and/or other staff. At the fourth site, the decision was made by the patient navigator with medical support where necessary.	(Site 4) Patient A was referred to the NSS pathway, and the GP referral said that the patient had recently undergone a colonoscopy in which four polyps were discovered. The patient had had a changed bowel habit for the last 5 weeks, and the GP was concerned. The team tried to decide what would be the most useful plan of investigation. Eventually, they contacted the consultant who did the patient’s colonoscopy for advice. They had to wait a while for an answer, but they felt it was safer than subjecting the patient to a CT scan straight away. The team also felt safer that the consultant was informed in case they wanted to perform a repeat colonoscopy.
To track patients’ reports and results	Once patients had been sent for tests and investigations, pathway navigators regularly checked whether their results and reports had been posted. Each site maintained a spreadsheet of patients so that they could monitor how many days had elapsed, and recall which investigations had been ordered. All sites used the patient record to access reports (eg, from radiology). There was some variation in terms of the use of the electronic patient record, with one site relying mainly on paper-based notes (diary) to note any key information for tracking, one site using email, and two sites using the patient record to note investigations, including flagging systems to indicate transfers of responsibility or other alerts.	(Site 2) Patient B spoke to the nurse about their upcoming CT scan and mentioned that they were also due for a regular colonoscopy. The nurse assured them that they would receive an appointment for the CT scan soon and agreed to follow up on the colonoscopy on the patient’s behalf so that it could be included in the NSS process. A week later, Patient B was still waiting to hear about their colonoscopy appointment. They decided to call the nurse again and ask about the status of their procedure. The nurse informed them that there was a huge backlog due to COVID, and assured them that they wouldn’t have to wait for too long. Patient B expressed frustration that they had to call the nurse four times already, and the nurse mentioned that she had already chased up the colonoscopy three times. Patient B felt that the nurse was not logical or thorough, and did not trust that their tests were being tracked effectively. Downstream, the nurse’s approach to tracking testing led to an incorrect investigation plan and suboptimal care.
To track other specialists advice or messaging	NSS staff communicated constantly with specialists to support their decision-making about investigations and interpretation of results. Variation occurred depending on the relationship of the medical and navigator staff to other specialties in the hospital. For example, good relationships were developed with some specialties if they had previously worked there or had regular contact. Other variations occurred in terms of the permeability of specialties; some services were open to giving informal advice, for example, over email or telephone. Others required formal transfer of tasks or a referral form.	(Site 1) Consultant C was preparing for the next patient, a vulnerable adult with potential cardiac issues. There were some concerns about heart failure, inflammation, or even malignancy. With serious cardiac issues, the consultant felt they couldn’t just see the patient in the NSS clinic. After some discussion, they decided to contact cardiology and see if they could help. Instead of emailing, they decided to try to visit the clinic and speak to the administrator in person to see what could be done due to their experience that cardiology could be unresponsive over email.After the clinic, Consultant C bumped into a colleague from cardiology in the corridor and asked their opinion on the patient. Impressively, Consultant C was able to remember all the patient’s test results on the spot. After some discussion, it was agreed that the patient should be seen by cardiology instead of the NSS team, and informal arrangements were made for a cardiac scan the next week. Consultant C seemed relieved and pleased with the decision, particularly the serendipity of bumping into their colleague in the corridor. Downstream, reliance on informal arrangements could have led to delays in the patient receiving appropriate investigations due to the need to go through multiple channels to get the patient to the right team.
To discuss the case with a multidisciplinary NSS team	Physical proximity was an important contextual factor which varied according to site. At three sites, the nursing and/or navigator team all sat together. At one site, the team was very small and sat in different offices. Two sites held regular MDTMs, with a third holding a weekly discussion if needed. The fourth team did not have a formal MDTM but spoke daily as they sat in proximity. They had a formal case-sharing for unusual cases as a learning event. MDTM case discussion was critical to retain the short-term memory of each patient, which facilitated faster and safer decision-making.	(Site 2) This is an example of a site without an MDTM. Consultant A was preparing for an upcoming phone call with the patient. He searched through his email notes but found nothing, so he turned to the referral form for information. The GP had noted something about ‘CaLung’, but it was unclear whether the patient had lung cancer. As he continued to search, he came across a chest X-ray report in the patient’s history. The report indicated that the patient had been diagnosed with chronic obstructive pulmonary disease, but there was no sign of lung cancer.The consultant concluded that the patient was simply at a high risk of developing lung cancer. He then looked through the assessment notes taken by the nurse but found them to be very brief and unhelpful. For example, the family history of cancer was simply listed as “father, brother, cousin, uncle”. Frustrated, the consultant remarked, ‘That doesn't help me much’. Upstream, the lack of MDTM meant that the consultant was not familiar with the patient and there were no notes about the team’s decision about how to investigate. Downstream, the lack of MDTM hindered decision-making and resulted in potentially inappropriate next steps.
To estimate remaining cancer risk	Following investigations, the NSS team had to evaluate whether any significant cancer risk remained. Variation was observed in how concerned NSS teams were by remaining symptoms and the extent to which they consulted patients about their preferences for ongoing testing. At all sites, a normal CT scan was interpreted as the strongest indicator of low cancer risk. Ongoing symptoms, such as weight loss or pain were often taken as a sign to pursue further tests. The remaining cancer risk was also weighed against any current or past GP investigations (indicated in the referral form) or patient preferences (eg, for minimal investigation).	(Site 4) Dr B had a complex patient who suffered from schizophrenia. Dr B had to determine the risk of a bowel thickening seen on the patient’s recent scan, but she was not experiencing any bleeding. Dr B knew they couldn't rule out cancer without a sigmoidoscopy, but the patient was not willing to undergo the invasive test. Dr B understood that pushing the patient would cause them to avoid future appointments. The other options for testing were an MRI or CT, but the patient had a fear of travelling and getting sick. Dr B decided to change the appointment to a telephone consultation, which would avoid the need for invasive testing. Dr B gave the patient some iron and found that she was iron-replete and polycythaemic. This was a reassuring sign, and Dr B suggested they wait and see what happens in a few months' time. Dr B was satisfied with the decision, knowing that the risk of cancer was low and that they had to find another way to manage the situation. Downstream, Dr B’s careful and considered approach to this patient’s care resulted in a safe and effective management plan that took into account the patient’s individual circumstances, concerns and preferences.
To communicate test results and formulation to patients	This function was typically carried out by either a patient navigator or a medical member of the team. Patient navigators often delivered results as they were reported and available on the electronic patient record. Conversely, medical staff delivered results and/or a formulation at the point of discharging or referring the patient, either in person or over a telephone appointment. Variation was observed in terms of whether the results were delivered in a scheduled clinic or as an unscheduled phone call.	(Site 3) A patient anxiously awaited their scheduled doctor’s call with the results of their recent tests. When the phone rang, the doctor began with good news, “everything was fine, no big problems.” Drawing on the patient’s investigation results and discussion with specialist colleagues, the doctor explained that they had found diverticula, which are common in older women. Additionally, there was an issue with the bones in their back, but nothing that couldn't be explained by old age. The doctor reassured the patient that they would write to their primary care physician to see if further action was necessary.The doctor recommended drinking lots of water and eating high-fibre foods and reassured the patient that following this advice would not make their diverticulitis worse. The patient was impressed with the efficiency and kindness of the medical team and was now at ease with their results. Downstream, this could have led to better patient satisfaction, improved compliance with treatment plans and more positive health outcomes. However, an unscheduled call like this could also have led to patients omitting questions or key concerns, leading to misunderstandings or missed opportunities for early intervention if a problem developed in the future.
To organise monitoring and surveillance of incidental findings	Variation was observed between sites in terms of willingness to carry out surveillance or follow-up. Two sites would not do any surveillance or follow-up. Two sites did surveillance depending on the protocol or expectations of other sites in the hospital. To some extent, this was determined by what other specialties are willing to do. There were concerns that this was a growing burden on the service with a lot of surveillance patients on the ‘books’ (but not on a cancer pathway).	(Site 4) Consultant C found it frustrating that some departments do not accept patients for surveillance, despite the fact that they have good electronic records that make the process relatively straightforward. When incidental results from scans or tests came up, they were responsible for requesting follow-up scans, which added a lot of extra work. Consultant C and their team handled incidental or abnormal findings, such as ovarian cysts, which require a year of surveillance. Downstream, the growing burden of surveillance has impacted the safety of all other pathway functions through reduced staff capacity.

GP, general practitioner; MDTM, multidisciplinary team meetings; NSS, non-specific symptom.

### Team composition and impact on pathway performance

We identified two background functions that contributed to safety and quality of care in NSS pathways. These functions relate particularly to managing the complexity of the overall functional system and the uncertainty of the patient’s underlying disease through the construction of a team capable of generalist and specialist medical work, underpinned by patient navigation.

#### Creating a generalist team with specialist skills to explore the uncertain underlying cause of patients’ symptoms

There was wide variation in team composition across the four sites, both in terms of the healthcare professions included and the size of the team. In all cases, the NSS service team had been designed explicitly to hold generalist knowledge that would enable high-quality investigations. This was achieved in different ways. For example, three sites had a single consultant in charge of the service who would lead some elements of triage and clinical decision-making. The consultant specialty varied, including radiology, gastroenterology and general medicine. In the fourth case, the navigator led triage, supported by medical input when necessary. In interviews, participants had broadly similar views about appropriate medical disciplines to lead an NSS service, choosing disciplines that were aware of a broad range of conditions and organ systems:

I am a gastroenterologist but also in general medicine, but I think, as a gastroenterologist we are probably exposed to quite a wide range of pathology and even without my general medicine hat on and probably a lot of things we see are what are factored pathways like cancer pathways, weight loss, difficulty swallowing, loss of appetite, anaemia, change in bowel, these are all, although they are GI symptoms, they are not necessarily specific symptoms for GI pathways. (RDC13 Consultant)

All sites had at least one nurse, who, in some cases, acted as a patient navigator. The nurses typically had a background in oncology. One site had radiographers who acted as patient navigators. Two sites had additional medical staff to support face-to-face clinic work but who did not lead triage and investigations. These included gerontologists and GPs who were recognised for their generalist knowledge in managing patients in the NSS pathway.

So what has kind of become apparent, and we’ve not studied this in any formal way, is that you need someone—like generalists, so whether that’s a GP—I don’t think it’s a discipline specific person. Because it is primarily abdominal you could be a gastroenterologist. But you could also be like a gerontologist, we don’t have that in (geographical region), but I know in other sectors they do. Like a very, an experienced GP. But like (Dr X) is an acute medical physician. (RDC01, Cancer Alliance)

Clinical participants described a wide range of knowledge, from elderly care medicine training, as well as knowledge of acute and chronic disease medicine. Several participants noted that a key factor in the quality of care was not just particular medical knowledge but also the ability to recognise when to refer to another specialty (see earlier section *Drawing on wide range of knowledge to formulate a diagnosis*).

Despite a range of relevant experience, many participants described the development of new skills specifically related to NSS work. These included interpretation of the filter function (blood) tests, interpretation and management of NSSs (eg, how to investigate weight loss), patient assessment techniques and risk threshold development and calibration (eg, when to discharge the patient). All sites had evolved activities to develop these skills, such as clinician-led training, multidisciplinary team meetings to discuss complex or unusual cases and peer-to-peer learning.

#### Creating continuity within a complex functional system through patient navigation

Many of the functions within the NSS pathway were performed by a patient navigator, with variation across the four sites. For example, some navigators screened GP referrals, accepted patients onto the pathway, organised testing and reported results to patients. In all sites, the navigator’s role was to manage the complexity of the functional system to create continuity. This included speaking with patients regularly to explain the management plan, coordinate testing and enable patient access. The service specification documents relating to the NSS pathway stipulated that ‘*Patients should be supported through their pathway with (as far as possible) a single point of contact sending SMS/email appointment reminders. This may be delivered by pathway navigator roles or existing members of the care team, with the approach tailored to suit the pathway*’*.* (NHS Cancer Programme Faster Diagnosis Framework). In three sites, the single point of contact concept was upheld. In a fourth site, multiple staff members engaged patients on the phone for assessments and clinic appointments.

Three of the four sites had patient navigators with clinical acumen, and this was seen to be an important driver of safety. In one site, with a non-clinical patient navigator, they were unable to relay test results on the phone, so this task fell to other members of the team, creating a discontinuity for the patient. One Cancer Alliance participant was adamant that the patient navigator should be a clinical role, in order to combine the ability to communicate with other clinicians and know the patient well:

The navigator has to sit within secondary care, they have to have clinical acumen so they have respect amongst their team, that’s why it can’t be a band 4 admin person. They have to be able to have sensible conversations out into primary care, so they have to be available and on the phone. They have to know the patient, so that means picking the patient up at the front door, having those conversations about don’t worry about your dog, someone will look after them on Thursday, we have a slot. (RDC19 Cancer Alliance)

Aside from medical knowledge and clinical skills, several participants reported that patient navigators needed the initiative to drive forward the wide range of investigations, including clinical decision-making. For example, a navigator may have to use initiative to spot when a different test than the one ordered would be more appropriate. Another aspect requiring initiative was making sure that the patient was cared for until a handover with another team was complete. For example, one navigator mentioned that they sometimes had patients who required immediate admission following their scan results and that they would ensure the handover was safe.

## Discussion

This study outlined the functions that enable NSS teams to investigate cancer and other diagnoses for patients with NSSs. The system model we produced outlined two clusters of foreground functions, within which we identified 10 that were safety-critical in NSS pathways. These included gathering historical patient health information, seeking advice from various specialties and continually assessing remaining cancer risk. The model exposed interdependencies and downstream effects, highlighting the importance of detailed, holistic note-taking in diagnostic decision-making and patient care. Background functions relating to team dynamics included forming a generalist team with specialist skills, fostering a learning environment for skill expansion, and managing functional system complexity through pathway navigation.

NSS pathways for cancer are now widespread in the UK, with similar developments in Sweden and Norway, as well as more developed services in Denmark. To our knowledge, this is the first study to address the safety of NSS pathway design in the UK; previously published studies have mainly been observational cohorts reporting diagnostic yield and patient characteristics.^7 13 15 21 22 26 28 30^ These studies have highlighted the significant heterogeneity of patients using NSS pathways^12 21 22 26^; our study highlights that this heterogeneity results in extensive ‘detective work’ for staff to decide on the most appropriate investigations for patients. This paper also builds on our prior publications, which focused on the experiences of older people with multiple concurrent concerns that are under investigation in NSS pathways and how they make sense of their care.^31^ We also explored policy implementation issues, including the role of NSS pathways in investigation conditions other than cancer.^38^

Our findings also highlighted similarities and variations in delivery and design. Previous research in Denmark, Norway, Sweden and the UK has established common testing strategies in NSS pathways^13 15 16 19 28^ as well as significant regional differences, particularly in roles and responsibilities for general practitioners and diagnostic units, preferential use of CT as a first-line investigation and primary care referral pathways.^13 19 29^ This study is a significant addition in terms of evidence about safety-critical functions; a particularly valuable contribution is around the end of the pathway and the functions relating to discharge, monitoring and further testing. A local NSS pathway evaluation reported that as many as 32% of patients receive further investigations after an initial negative scan.^39^ The decision to order multiple tests is a concern for overdiagnosis, patient safety and cost and should be the subject of future research.

Finally, our background functions relating to team dynamics reveal novel challenges for cancer pathway organisation. Our findings relating to generalist-specialist skill development suggest that multidisciplinary team discussion is valuable in cancer pathways for patients with non-site-specific symptoms and having more than one consultant per team would improve safety. Our results also suggest that patient navigators should have a clinical background (eg, nursing or radiography) to enable better continuity, initiative taking and patient communication. Previous research suggests that patient navigator roles and close monitoring are highly valued and support equality of access.^28 40 41^ Future research should focus on different models of generalist-specialist team composition and skill development, particularly in view of the likely development of multicancer early detection tests in symptomatic and asymptomatic populations.^42 43^ This should include studies using case studies, ethnographic and other methodological paradigms that can capture whole systems and map service design against a range of diagnostic outcomes.^44^ Additional targets for future research include the development of evidence-based guidelines for investigations, optimal systems to capture detailed patient information and communication strategies to support safe discharge in the presence of persistent symptoms.

### Limitations

By trying to capture the functions of a whole pathway across four sites, we have had to abstract some of the functions to retain usability in the resulting system model. This may have lost some of the nuance and specific workings of individual sites. Individual FRAM models could have been produced to capture specificity before identifying a communal model. However, we contend that our process and model assist the generalisability and utility of the visualisation of the functional system. Our model could be used as a template for other NSS services to personalise for their local environment. This would save time and resources for local improvement and research. Furthermore, we did not conduct our analyses fully in line with FRAM as described by Hollnagel,^35^ for example, by describing all six aspects of each function or by using the FRAM model visualiser. This may mean that we lost opportunities to understand key interdependencies between the functions.

## Conclusions

NSS pathways have been developed in a number of European countries to tackle the problem of delayed, fractured approaches to cancer diagnosis. This study is the first approach to describe how this is achieved in practice drawing on fieldwork capturing a range of practices and perspectives across different sites. We identified certain functions that are critical to patient safety and should be considered in further detail to optimise service quality and safety. Significant variation in these functions was identified within and between sites, and traditionally, approaches to improve safety have broadly sought to reduce variation (eg, through standardisation). However, this analysis has demonstrated that different sites are able to deliver safe care in different ways. National and local improvement plans could differentiate between unwarranted variation, where improvement may be supported through more specific policies or guidelines and adaptive variation, which should be supported and harnessed to facilitate the delivery of safe care within the local context. We have demonstrated that activities that foster learning, continuity and generalist-specialist skill development are highly valued and should be prioritised. At both national and local levels, a blueprint for service improvement may, therefore, focus on the specific safety-critical functions identified, provide recommendations on function (ie, aim) as well as form (ie, how this aim is achieved) and support cultural improvements, such as team dynamics that influence care delivery more broadly.

## Supplementary material

10.1136/bmjqs-2024-017749 online supplemental file 1

## Data Availability

Data are available upon reasonable request.
